# Medical Expert Testimony

**Published:** 1897-11

**Authors:** 


### EDITORIAL.

---

he office of the Business Manager of THE JOURNAL is 311 and 312 Fitten Building.

The editors are not responsible for views expressed by contributors.

Articles for publication should reach this office not later than the 15th inst.

Address all communications, and make all remittances payable to THE ATLANTA MEDICAL AND SURGICAL JOURNAL, Atlanta, Ga.

Reprints of articles will be furnished, when desired, at a small cost. Requests for the same should always be made *on the manuscript.*

---

### MEDICAL EXPERT TESTIMONY. THE ABUSE AND THE REMEDY.

Of late considerable thought and attention has been bestowed, by both lawyers and docters, upon the present unfortunate methods of obtaining and utilizing medical expert testimony, and many suggestions have been proposed as a means of eliminating the objectionable features which now exist.

That these abuses do exist is well known to any one who has had any experience in the modern court-room. It is known and commented upon even by the lay reader of the daily papers, to whom is not unfrequently presented the undignified spectacle of one or more medical men swearing one thing and one or more equally capable men swearing exactly the opposite. This is so common a picture that it is almost a court axiom that medical expert testimony is no testimony; and indeed, in many cases in which the conflict of expert opinion serves only to confuse the minds of the jury, it is easy to see how such testimony may be worse than useless and even complicate the ends of justice. Theoretically, the medical expert's function in the court-room is to aid the court and the jury in reaching just conclusions upon certain technical features of the case. Practically, both sides have their experts, and by the time the examinations and cross-examinations are over the last state of that court and jury is worse than the first. So, in the presence of such confusion worse confounded, it is not uncommon for juries to reject absolutely the conflicting testimony of the experts and to arrive at their verdicts from the other evidence. The mortifying circumstances which attend the giving and taking of medical expert testimony never had a better illustration than in the late trial of Leutgert in Chicago, in which certain experts identified certain bones as positively human (and probably those of a woman), and certain other experts just as positively swore that they were the bones of a hog or dog. (It was assumed, of course, that there was nothing remarkable about the bones of a dog being found in the vats of a sausage factory.) Justice certainly received no help from the learned experts in this case.

At present the opposing counsel select their experts, sometimes even paying retaining fees, and the medical men, consciously or unconsciously, adapt themselves as best they can to the requirements of their respective positions. Doctors are human, very human, and in their examinations of a given case and in their conduct upon the stand it is hard for them to get away from the fact that they appear in the interest of the side that employs them. Dr. Mettler, of Chicago, in a late paper, says: "When experts are thus hired they are almost forbidden to be experts by being made partizans. . . Thus the court-room [becomes] the scene of a scientific debate much to the dismay and confusion of an uninformed jury. The counsel fixes the medical opinions for the court and then hires those experts, or self-styled experts, who will be most likely to support his side of the contest. Between the two sides a rhetorical display of scientific quibbling is presented for the edification of the court and jury; more often a roaring farce is performed, the judge becomes incensed, the jury falls into hopeless confusion, and the few deserving experts in the case are brought to shame and made the victims of most unjust sarcasm. *Experts paid by the court or government should be employed, and only such experts.** That would be one step toward the elimination of the [illegible]izanship feeling."

It seems to be generally understood that the remedy just suggested is the proper solution of the abuses as they now exist. Mr. Robert Mather, of Chicago, general attorney for an important railway system, read an exceedingly able paper on this subject at the meeting of the American Academy of Railway Surgeons last year, from which we will select a paragraph:

"The remedy for the apparent abuses of expert testimony lies in the designation by the court in each case of the experts who shall be called to testify. There is no novelty in this suggestion; it has been frequently and ably urged, and I believe already commends itself to the honest judgment of both our professions. It will be observed, however, that I have used the term *designation* rather than *selection* by the court. If we always and everywhere had a judiciary that was able, impartial and strictly just, from which prejudice, partizanship and demagoguery were absolutely removed, I should favor the sole selection of experts by the judges. . . But there are tribunals in which both the parties and the blind goddess herself would prefer that the experts whose opinions are to guide the jury to a correct conclusion should be selected by the litigants. The parties, therefore, should be given an opportunity to agree, the court to make the selection in case of disagreement. But whether agreed upon by the parties or selected by the judge the experts should receive their appointment from the court. They should be officers of the court, sworn and acting as its commissioners for the better ascertainment of the truth. This plan would

* Italics ours.—ED.

remove the root of existing evils, the retainer system. The cause of the discredit in which expert testimony is now held being thus removed, the effect would disappear. The expert would be restored to his ancient and legitimate function of adviser to the court and jury, and in this character would win back his place in the confidence of the courts and the community."

The necessity for suitable legislation to correct these evils has been thoroughly pointed out in several important papers in the last two or three years, and steps have been taken in this direction in several States. The profession in Georgia is interested in this matter. At the late meeting of the Association in Macon, a Committee was appointed to take the subject in hand, to prepare a suitable bill and to have it introduced at the next meeting of the legislature. The Chairman of the Committee tells us that this bill has been drafted, and that it will be pushed with vigor in the legislature.

### PROGRESS OF THE YELLOW FEVER.

Following is a statement of the Yellow Fever record to date (October 24th), compiled from the Associated Press dispatches:

| | CASES | DEATHS |
|---|---|---|
| New Orleans, La | 1,115 | 129 |
| Ocean Springs, Miss | 797 | 11 |
| Edwards, Miss | 510 | 30 |
| Biloxi, Miss | 503 | 23 |
| Scranton, Miss | 259 | 10 |
| Mobile, Ala | 209 | 28 |
| Flomaton, Ala | 36 | 0 |
| Montgomery, Ala | 56 | 4 |
| Wagar, Ala | 9 | 1 |
| St. Elmo, Ala | 5 | 0 |
| Franklin, La | 5 | 0 |
| Scattering | 44 | 9 |
| Total | 3,548 | 245 |

This represents a mortality of about seven per cent., which indicates a somewhat milder type of the disease than in the usual epidemic. In the meantime it does not seem possible to stamp out the disease in places where it has gained a foothold. Frost is eagerly awaited as the only certain check to the progress of the fever. Fortunately it cannot be far off.

It is announced that Sanarelli, the discoverer of the bacillus of yellow fever, has just perfected a curative serum, and that he will soon publish the results of his experiments.

Dr. Guiteras, the well-known authority on yellow fever, who has visited all the infected places, says: "The source of this epidemic is, as usual, the island of Cuba. At least 90 per cent. of the cases of yellow fever imported into the United States from which epidemics have resulted have been introduced from Cuba. The last great epidemic was that of 1878. It cost this country many thousands of lives and $200,000,000. That amount, added to what the present affliction will entail, would be more than enough to buy the whole island. A complete system of sanitation in Havana is the only means to prevent importing the disease into the United States. American methods of sanitation could eradicate it from Havana. The attitude of the Spanish government with regard to the malady has always been that of criminal indifference."

---

WE are in receipt of the Transactions of the American Academy of Railway Surgeons for the third annual meeting, Chicago, September 23d-25th, 1896. The volume is edited by Dr. R. Harvey Reed, Secretary, Columbus, Ohio. It contains many excellent papers, the greater part of which relate to railway surgery. The address of President Owens and the paper of Mr. Mather, both on Medical Expert Testimony, are particularly noteworthy.

